# Investigation Into the use of  C- and N-terminal GFP Fusion Proteins for Subcellular Localization Studies Using Reverse Transfection Microarrays

**DOI:** 10.1002/cfg.405

**Published:** 2004-06

**Authors:** Ella Palmer, Tom Freeman

**Affiliations:** MRC Rosalind Franklin Centre for Genomics Research (formerly the HGMP-Resource Centre) Genome Campus Hinxton Cambridge CB10 1SB UK

## Abstract

Reverse transfection microarrays were described recently as a high throughput method for studying gene function. We have investigated the use of this technology for determining the subcellular localization of proteins. Genes encoding 16 proteins with a variety of functions were placed in Gateway expression constructs with 3′ or 5′ green fluorescent protein (GFP) tags. These were then packaged in transfection reagent and spotted robotically onto a glass slide to form a reverse transfection array. HEK293T cells were grown over the surface of the array until confluent and GFP fluorescence visualized by confocal microscopy. All C-terminal fusion proteins localized to cellular compartments in accordance with previous studies and/or bioinformatic predictions. However, less than half of the N-terminal fusion proteins localized correctly. Of those that were not in concordance with the C-terminal tagged proteins, half did not exhibit expression and the remainder had differing subcellular localizations to the C-terminal fusion protein. This data indicates that N-terminal tagging with GFP adversely affects the protein localization in reverse transfection assays, whereas tagging with GFP at the C-terminal is generally better in preserving the localization of the native protein. We discuss these results in the context of developing high-throughput subcellular localization assays based on the reverse transfection array technology.


**Comparative and Functional Genomics**
*Comp Funct Genom* 2004; **5**: 342–353.
Published online in Wiley InterScience (www.interscience.wiley.com). **DOI:** 10.1002/cfg.405

**Research Paper**

### Investigation into the use of C- and N-terminal GFP fusion proteins for subcellular localization studies using reverse transfection microarrays

Ella Palmer and Tom Freeman*
*MRC Rosalind Franklin Centre for Genomics Research (formerly the HGMP-Resource Centre), Genome Campus, Hinxton, Cambridge CB10 1SB, UK*

**Correspondence to:*
*Tom Freeman, RFCGR, Hinxton, Cambridge/CB10 1SB, UK.*
*E-mail: tfreeman@rfcgr.mrc.ac.uk*

Received: 6 October 2003
Revised: 5 March 2004
Accepted: 5 March 2004

### Abstract

**Reverse transfection microarrays were described recently as a high throughput method for studying gene function. We have investigated the use of this technology for determining the subcellular localization of proteins. Genes encoding 16 proteins with a variety of functions were placed in Gateway expression constructs with 3′ or 5′ green fluorescent protein (GFP) tags. These were then packaged in transfection reagent and spotted robotically onto a glass slide to form a reverse transfection array. HEK293T cells were grown over the surface of the array until confluent and GFP fluorescence visualized by confocal microscopy. All C-terminal fusion proteins localized to cellular compartments in accordance with previous studies and/or bioinformatic predictions. However, less than half of the N-terminal fusion proteins localized correctly. Of those that were not in concordance with the C-terminal tagged proteins, half did not exhibit expression and the remainder had differing subcellular localizations to the C-terminal fusion protein. This data indicates that N-terminal tagging with GFP adversely affects the protein localization in reverse transfection assays, whereas tagging with GFP at the C-terminal is generally better in preserving the localization of the native protein. We discuss these results in the context of developing high-throughput subcellular localization assays based on the reverse transfection array technology. Copyright © 2004 John Wiley & Sons, Ltd.**

**Keywords:** **reverse transfection; subcellular localization; signal sequence; C- and N-terminal tag; cell-based array**

### Introduction

Reverse transfection (Ziauddin, 2001) is a powerful new technology with the potential to provide a high throughput screen of gene function, identification of novel drug targets and determination of the subcellular localization of proteins (Wu, 2002; Bailey, 2002). Reverse transfection technology entails inserting full-length open reading frames (ORFs) of genes of interest (GOI) into an expression vector. These vectors are then packaged into a transfection reagent and printed onto a glass slide to form a microarray. Cells are grown over the top of the array until confluent and patches of transfected cells overexpressing the genes are formed (Figure 1). Arrays can then be examined for alterations in cellular function as manifested in changes to the biochemistry or morphology of cells. If the expression vector contains a 'tag', the subcellular localization of the transgene can also be analysed. Using this approach, large numbers of assays can be performed in a single study.

In order to tag proteins for determination of their localization, two basic approaches are utilized for detection; epitope tagging, or tagging of the GOI with a protein that is fluorescent or possesses enzymatic activity. Epitope tags are small peptides

Copyright © 2004 John Wiley & Sons, Ltd.

(3–14 amino acids), e.g. $His_6$ and c-myc, which can be detected by immunohistochemistry. Due to their small size, they are less likely to disrupt the normal function or localization of the protein. However, immunohistochemical detection is time-consuming, and processing can be disruptive to the layer of cells growing over the array and can only be performed on fixed cells. Alternatively, protein tags such as luciferase, $\beta$-galactosidase ($\beta$-gal) and green fluorescent protein (GFP) can be detected through their innate activity. GFP has the advantage that it is stable for months, can be visualized easily via standard confocal or fluorescent microscopy (Lippincot-Schwartz, 2003), is readily available in expression vectors such as the Invitrogen Gateway cloning system and can be detected in living cells.

We wished to investigate the use of GFP to study the subcellular localization of proteins following reverse transfection. In particular, we sought to study the effect of positioning GFP at either the N- or C-terminal on the subcellular localization of the fusion protein — if this is disrupted relative to the native protein, so presumably will be the normal functional activity of the protein. In order to establish a working protocol for reverse transfection and explore its potential as a high-throughput screen of subcellular localization, we selected 16 genes from three functional classes; kinase, transcription factor and surface receptor. In addition, the genes were selected because they were of a range of sizes and were known to localize to differing subcellular compartments (recorded by Swiss-Prot). The Mammalian Gene Collection (MGC; Strausberg, 2002) was chosen as a library from which to obtain the genes as it contains a large number of full-length, sequence-verified open ORFs. The genes were amplified and placed into Gateway destination N- and C-terminal GFP fusion vectors. Using the reverse transfection strategy, these constructs were transfected into HEK293T (human embryonic kidney) and the subcellular localization of the transgene recorded. These observations were correlated with the documented and/or predicted localizations for these proteins, allowing the effect of tagging to be examined.

### Materials and methods

### Amplification of gene of interest (GOI) open reading frames (ORF)

Sequence verified open reading frames (ORFs) in the vector pCMV-SPORT6 (Invitrogen, Paisley, UK), were obtained from MRC gene-services (HGMP-RC, UK), which are part of the IRAT set from the MGC collection. Replicate working plates were prepared in LB media containing 8% glycerol (Sigma, Dorset, UK) and 50 µg/ml ampicillin (Sigma), grown at 37 °C overnight and stored at −70 °C. 5′ primers were designed with CACC (to facilitate insertion of the GOI into the Gateway entry vector pENTR/D-TOPO) plus a further 18 bp from the first bp of the start codon. 3′ primers were designed with 18 bp before the first bp of the stop codon (in order that transcription can continue through the gene to the stop codon of the C-terminal GFP). The melting temperature ($T_m$) of the primers was calculated using the formula: $[6.93 + 0.41(\%G + C)]$ — (650/l) (Chester, 1993). 55–60 °C was the ideal for each primer pair; if necessary, bases were added or removed to compensate and the primer pairs were checked for non-complementarity using a Jemboss programme, Water; **http://www.hgmp.mrc.ac.uk/Software/EMBOSS/Jemboss**. For the sequence of all primers used in this study, see Table 1. A 20 µl aliquot of the IRAT clone culture from the replicate plates was added to 180 µl water to lyse the *Escherichia coli* cells and 3 µl of each clone dilution was added to a PCR reaction mix containing: 2.5 µl 10× amplification buffer (provided with *Pfx* polymerase), 0.75 µl 10 mM dNTP, 0.5 µl 50 mM $MgSO_4$, 100 ng 5′ primer, 100 ng 3′ primer mix, 0.3 µl 2.5 U/µl *Pfx* polymerase (Invitrogen, Paisley, UK) and made up to 25 µl with $H_2O$. A control reaction was set up as above with primers to the pCMV–SPORT-6 vector either side of the GOI. If reactions failed, they were repeated with an annealing temperature of 55 °C. Amplified ORFs were purified using the Qiaquick PCR purification kit (Qiagen); the correct sizes were confirmed using an Agilent Bioanalyzer 2100 (Agilent, West Lothian, UK).

### Transfer of amplified ORFs into pENTR/D-TOPO clones pENTR/D-TOPO is an entry vector for the Gateway cloning system. It is supplied linearized with a GTGG overhang end and a blunt end to allow the GOI ORFs with a CACC overhang to insert in one direction only. The 16 GOIs and a control were inserted into pENTR/D-TOPO and then transformed in One Shot TOP10 *E. coli* cells, according to the manufacturer's instructions (Invitrogen);

Copyright © 2004 John Wiley & Sons, Ltd. 

**Table 1.** Forward and reverse primers for amplification of ORFs from IRAT clones in pCMV-SPORT6 vectors

| Gene | Primers | Sequence | Gene | Primers | Sequence |
|---|---|---|---|---|---|
| ATF4 | Forward | 5′-caccatgaccgaaatgagcttc-3′ | NFIL3 | Forward | 5′-caccatgcagctgagaaaaatg-3′ |
| | Reverse | 5′-ggggacccttttcttccc-3′ | | Reverse | 5′-cccagagtctgaagcaga-3′ |
| CALM2 | Forward | 5′-caccatggctgaccaactgact-3′ | PPARG | Forward | 5′-caccatgaccatggttgacaca-3′ |
| | Reverse | 5′-ctttgctgtcatcatttgtacaaactc-3′ | | Reverse | 5′-gtacaagtccttgtagatctc-3′ |
| CDK7 | Forward | 5′-caccatggctctggacgtgaag-3′ | PTPN11 | Forward | 5′-caccatgacatcgcggagatgg-3′ |
| | Reverse | 5′-aaaaattagtttcttgggcaatcc-3′ | | Reverse | 5′-cctgcagtgcaccacgac-3′ |
| CDK9 | Forward | 5′-caccatggcgaagcagtacgac-3′ | SIP2-28 | Forward | 5′-caccatggggggctcgggcagt-3′ |
| | Reverse | 5′-gaagacgcgctcaaactcc-3′ | | Reverse | 5′-caggacaatcttaaaggagctg-3′ |
| CDKN1B | Forward | 5′-caccatgtcaaacgtgcgagtg-3′ | STK15 | Forward | 5′-caccatggaccgatctaaagaa-3′ |
| | Reverse | 5′-cgtttgacgtcttctgaggc-3′ | | Reverse | 5′-agactgtttgctagctgattc-3′ |
| CXADR | Forward | 5′-caccatggcgctcctgctgtgc-3′ | TGIF | Forward | 5′-caccatgaaaggcaagaaaggt-3′ |
| | Reverse | 5′-tactatagacccatccttgct-3′ | | Reverse | 5′-agctgtaagttttgcctgaag-3′ |
| IL17BR | Forward | 5′-caccatgtcgctcgtgctgcta-3′ | TNFRSF10B | Forward | 5′-caccatggaacaacggggacag-3′ |
| | Reverse | 5′-caaggagcagcagccatc-3′ | | Reverse | 5′-ggacatggcagagtctgca-3′ |
| MARKL1 | Forward | 5′-caccatggcagctctgcgccag-3′ | SP6 (pCMV-SPORT6) | Reverse | 5′-atttaggtgacactatag-3′ |
| | Reverse | 5′-gagctcgaggtcgttgga-3′ | M13 (pENTR/D-TOPO) | Forward | 5′-gtaaaacgacggcc-3′ |
| NFIB | Forward | 5′-caccatgatgtattctcccatc-3′ | T7 (CMV-SPORT6/ pcDNA-DEST47+53) | Forward | 5′-taatacgactcactataggg-3′ |
| | Reverse | 5′-gcccaggtaccaggactg-3′ | | | |

Primers were designed with a CACC overhang at the 5′ end to facilitate gene insertion into the Gateway pENTR/D-TOPO entry vector and also to remove the stop site from the GOI for subsequent fusion with the C-terminal GFP.

they were then spread onto 50 μg/ml kanamycin (Sigma) 2XTY agar plates and incubated at 37 °C overnight. Five colonies were picked for each gene and cultured overnight in 5 ml LB medium containing 100 μg/ml kanamycin. 15% glycerol stocks were prepared with 1 ml of the overnight culture and the remainder was purified with the Concert plasmid miniprep system, according to the manufacturer's instructions (Invitrogen).

### Ligation, propagation and linearization of destination vectors pcDNA-Dest53 and 47 are destination vectors for Invitrogen's Gateway cloning system and form fusion genes with GFP at the 3′ or 5′ end, respectively. They are supplied linearized, and so were ligated prior to propagation. The ligation reaction contained 150 ng pc-DNA-Dest47 or 53, 1 U T4 DNA ligase (Roche, Basel, Switzerland), 4 μl 10× ligase buffer and was made up to 40 μl with $H_2O$. The reaction was incubated at room temperature for 3 h. The DNA from the ligation reactions was diluted five-fold in 10 mM Tris–HCl (pH 7.5) and 1 mM EDTA, 1 μl (10 ng DNA) of the dilution was added to DB3.1-competent *E. coli* cells (Invitrogen), which are resistant to the ccdB gene contained in pcDNA-Dest47 and 53 before recombination. 150 μl was plated onto a 100 μg/ml ampicillin LB plate and incubated overnight at 37 °C. 10 colonies were picked and grown up overnight in 5 ml LB medium containing 100 μg/ml ampicillin (Sigma). For the most efficient recombination with the entry clone, the destination vector needs to be linear, therefore a restriction digest was prepared with 3 μl 10 × buffer, 0.3 μl 10 mg/ml BSA, 1 μg pcDNA-Dest47 or 53 and made up to 30 μl with $H_2O$. 0.75 μl *Eco*R1 (Promega) was added and the digest incubated at 37 °C for 1 h.

**Figure 1.** Reverse transfection strategy. (1,2) The genes of interest (GOI) were amplified from MGC clones in the pCMV-SPORT6 vector. Primers were designed with a CACC overhang at the 5′ end to facilitate gene insertion into the Gateway pENTR/D-TOPO entry vector and also to remove the stop site from the GOI for subsequent fusion with the C-terminal GFP. (3) Once in the entry vector the gene was recombined into Gateway destination vectors pcDNA–Dest47 (C-terminal fusion GFP) and pcDNA–Dest53 (N-terminal fusion GFP). (4,5) The vector was packaged in Effectene transfection reagent and spotted onto a glass slide in a 10 × 10 grid (purple squares indicate C-terminal GFP fusions and orange squares N-terminal fusions). (6) HEK293T cells were grown over the array to a confluent monolayer. (7) Patches of transfected cells and the subcellular localizations of the expressed GFP fusion proteins were visualized by confocal microscopy

Copyright © 2004 John Wiley & Sons, Ltd.

### Recombination of pENTR/D-TOPO and pcDNA-Dest47 and 53 (LR reaction)

The LR reaction was prepared with 4 µl LR reaction buffer, 300 ng pENTR/D-TOPO containing GOI, 300 ng pcDNA-dest47 or 53, 4 µl LR clonase enzyme mix and made up to 20 µl with TE buffer. It was incubated at 25 °C for 60 min, 2 µl proteinase K (Invitrogen) was added to stop the reaction and then incubated at 37 °C for 10 min. The reactions were transformed in DH5$\alpha$ cells according to the manufacturer's instructions (Invitrogen). 10 µl and 100 µl were plated onto 100 µg/ml ampicillin plates and incubated overnight at 37 °C. Three colonies from each gene were picked and incubated overnight at 37 °C in 5 ml LB medium containing 100 µg/ml ampicillin. Glycerol stocks were made and the plasmid purified as for pENTR/D-TOPO vectors.

### Confirmatory PCR

### pENTR/D-TOPO

A PCR reaction was set up for each GOI, positive and negative (no DNA) controls, containing 2 µl 10 × buffer, 0.5 µl 10 mM dNTP, 1 µg pENTR/D-TOPO vector containing GOI, 0.2 µl *Taq* polymerase, 100 ng 5′ vector specific M13 primer (Table 1) 100 ng 3′ gene specific primer (Table 1), and made up to 20 µl with $H_2O$. PCR cycling was performed as follows: 94 °C 15 min; then (94 °C 1 min, 55–60 °C 2 min, 72 °C 7 min) ×30 cycles; finally 72 °C 10 min. Annealing temperatures were dependent upon empirical determination of the optimum PCR primer conditions.

### pcDNA-Dest 47 and 53

Confirmatory PCR was carried out as for pENTR/D-TOPO with the 5′ vector primer T7 (Table 1) and the gene-specific 3′ primer (Table 1). The correct sizes of the amplified GOI ORF were confirmed using an Agilent Bioanalyzer 2100.

### Reverse transfection

Destination vectors containing the GOI were prepackaged in the transfection reagent Effectene (Qiagen) prior to printing. 1 µg pcDNA-Dest47 or 53 containing the GOI was added to 15 µl DNA condensation buffer. 1.5 µl enhancer solution was added and incubated at room temperature for 5 min. 5 µl Effectene was added, the solution mixed with gentle vortexing, incubated at room temperature for 10 min and a 1× volume of 0.05% gelatin (Sigma) added. Samples were printed with a Biorobotics MicroGrid II microarrayer onto polylysine slides (Sigma) in a 10 × 10 square, the pins hitting each spot twice. Each spot was ∼140 µm in diameter. Arrays were stored desiccated at room temperature. Human embryonic kidney (HEK293T) cells were grown and maintained in 500 ml DMEM with 0.11 g/l NA PYR with pyroxidine containing 50 ml FCS, 100 U/ml penicillin, 100 µg/ml streptomycin (Invitrogen) at 37 °C and 5% $CO_2$. $10 \times 10^6$ cells were added to 20 ml culture medium and inverted four times to mix. Three slides were placed in a 10 × 10 cm square dish (Falcon) and the cell suspension poured onto the slides. The dish was placed in a 37 °C, 5% $CO_2$ incubator for 40 h, or until the cells were completely confluent.

The slides were rinsed in PBS (Gibco) for 10 s, fixed in 3.8% paraformaldehyde (38% paraformaldehyde (BDH), diluted to 3.8% with PBS) for 20 min, then dipped briefly in water to rinse. A drop of mounting medium containing propidium iodide stain (Vector) was applied to a coverslip (Amersham) and lowered onto the slide. Fluorescence was visualized using a Nikon Eclipse E800 microscope with a BioRad confocal attachment.

### Standard transfection

$5 \times 10^6$ HEK293T cells in 2 ml culture medium (as reverse transfection) were poured over circular 22 mm diameter cover slips (BDH) in six-well plates 24 h before transfection. 0.4 µg destination vector containing the GOI was added to 100 µl buffer EC (Qiagen) and 3.2 µl enhancer, vortexed and incubated at room temperature for 5–10 min. 10 µl Effectene was added, vortexed and incubated at room temperature for 5–10 min and 600 µl culture medium was added. Media was aspirated from the six-well plate, 1.2 ml fresh media added and the DNA transfection mix carefully pipetted over the cells. The plates were placed in a 37 °C, 5% $CO_2$ incubator for 40 h, or until the cells were completely confluent. The cells on the cover slips were fixed, stained and imaged as for the reverse transfection.

Copyright © 2004 John Wiley & Sons, Ltd.

### Database searches

The sequences of the 16 genes used in this study were analysed using the subcellular localization prediction programmes PsortII (**http://psort.nibb.ac.jp**), ProtComp 4 (**http://www.softberry.com**) and the signal peptide prediction programme PredictNLS (**http://cubic.bioc.columbia.edu/predict NLS**).

Information on previous subcellular localization studies and signal peptides was also obtained from Swiss-Prot/TrEMBL (**http://ca.expasy.org**) via the gene name.

### Results

Sixteen genes were chosen from three different functional classes; kinase, transcription factor and surface receptor, with differing subcellular localizations to ensure that the findings were widely applicable. The size and orientation of the gene insertions were successfully validated via PCR (data not shown). Protein expression was observed in all 16 of the C-terminal genes and 11 of the 16 N-terminal genes printed on the array.

Ziauddin and Sabatini (2001) reported a transfection efficiency of 30–80 cells/spot on their arrays with a spot size of 120–150 μm. In the current study, each gene was spotted in a $10 \times 10$ grid and the spots were approximately 140 μm in size. Transfection efficiencies of up to 30 cells/spot were observed; however, this varied greatly depending on the identity of the transgene. All the C-terminal tagged proteins except one had better transfection efficiencies than N-terminal tagged proteins. Nine C-terminal-tagged proteins (CXADR, TNFRSF10B, MARKL1, CDK9, TGIF, IL17BR, CALM2, NFIB and CDKN1B) showed transfection in 80% or more spots, with 10–30 cells transfected per spot. Five C-terminal tagged proteins (SIP2-28, NFIL3, PTPN11, PPARG and CDK7) showed transfection in 30–80% of spots, with 5–10 cells transfected per spot. Finally, two C-terminal tagged proteins, ATF4 and STK15, showed transfections in only 5–30% of their spots, with 2–5 cells transfected per spot. N-terminal tagged proteins showed transfection in 2–40% of their spots, with 2–10 cells transfected per spot. The only protein to show better transfection efficiency when tagged at the N-terminal was CDK7.

Of the 11 N-terminal tagged proteins expressed, only six fusion proteins (ATF4, CALM2, CDK7, CDK9, IL17BR and NFIB; Figure 2 and Table 2) had the same subcellular localization as the C-terminal fusions and are henceforth referred to as group 1. Five of the N-terminal fusion proteins (PTPN11, TNFRSF10B, TGIF, MARKL1 and SIP2-28) had differing subcellular localizations to the C-terminal fusion proteins (group 2; Figure 3). Five of the N-terminal fusion proteins (CXADR, NFIL3, PPARG and STK15 and CDKN1B) showed no transfection events at all (group 3; Figure 3). These results are summarized in Table 2.

All 32 transfections — 16 C-terminal and 16 N-terminal — were also undertaken using standard transfection techniques in six-well plates. The results were identical; group 1 proteins showed the same subcellular localizations whether tagged at the N- or the C-terminal. Group 2 proteins had the same localizations as recorded in the reverse transfection, with differing subcellular localizations depending on whether they were tagged at the C- or N-terminal. Group 3 proteins showed the same subcellular localizations at the C-terminal as the reverse transfection and no localization signal at the N-terminal (data not shown).

Swiss-Prot/TrEMBL (Boeckmann, 2003) record subcellular localizations from the scientific literature and 12 of the 16 proteins here have been studied previously. In seven cases the C-terminal localizations in this study were identical to the Swiss-Prot/TrEMBL entries; ATF4, CDKN1B, NFIB, PPARG and STK15 were confirmed to be nuclear, PTPN11 was confirmed to be cytoplasmic and CXADR was confirmed as plasma membrane. Of the remaining five C-terminal subcellular localizations, all were similar to Swiss-Prot/TrEMBL. Our subcellular localizations for CDK7, CDK9 and TGIF were the nucleus and one other organelle, whereas Swiss-Prot/TrEMBL reported the localization to be nucleus only. Previous studies could have been carried out in different cell types which can make a difference to the localization pattern. IL17BR and TNFRSF10B had been recorded as localizing to the plasma membrane, we found IL17BR in the endoplasmic reticulum and TNFRSF10B in tubules and vesicles within the cell.

Two bioinformatic prediction programmes were used to help support the localizations of proteins in groups 1 and 3 and to determine which

Copyright © 2004 John Wiley & Sons, Ltd. 

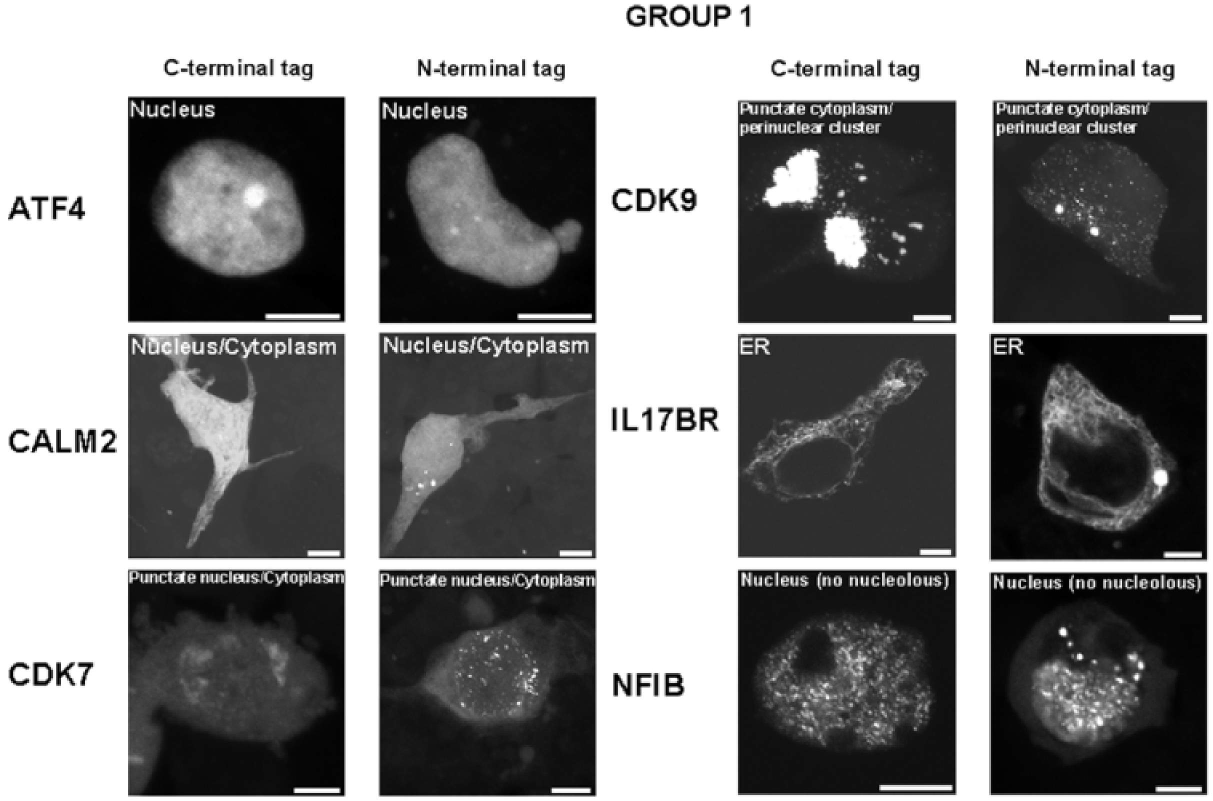

**Figure 2.** Group 1 C- and N-terminal tagged proteins with the same subcellular localization. A confocal microscope with a ×60 oil lens was used to obtain 16 slices through the cell for each image. All images are of the whole cell except proteins NFIB and IL17BR, which are slices to demonstrate the empty nucleus in IL17BR and the empty nucleoli in NFIB. Bar, 10 μm subcellular localization was more likely to be correct for the group 2 proteins. Psort II and ProtComp version 4 (Table 2) were chosen because they integrated different prediction methods to give a combined result. Psort II combines known protein sorting signals and amino acid compositions (Nakai, 1991, 1992, 1999) and then employs the $k$-nearest-neighbour method, which uses the results as a feature vector to calculate Euclidean distances between proteins (Nakai, 2001). Each organelle was given a subcellular localization likelihood score between 1% and 100%; results over 20% were recorded in this analysis.

ProtComp version 4 (Softberry) combines results with proteins of known subcellular localization and assumed subcellular localization (based on theoretical evidence) and provides a score assigned by neural networks. Each organelle was assigned a subcellular localization likelihood score between 0 and 10; scores over 1 are recorded in Table 2. These two programmes were chosen because they predict a variety of organelle localizations, whereas other prediction programmes provide a more limited readout. At least one of the two subcellular prediction programmes was in accordance with the observed C-terminal subcellular localization for each of the genes.

Four subcellular localizations, for NFIL3, CALM2, MARKL1 and SIP2-28, were determined that were not recorded by Swiss-Prot/TrEMBL. NFIL3 was localized to the nucleus with the C-terminal tag; the two bioinformatics programmes also predicted the nucleus. MARKL1 was localized to the mitochondria with the C-terminal tag, which was predicted in both programmes. CALM2 was localized to the nucleus/cytoplasm with both the N- and C-terminal tags, which the two programmes also predicted. SIP2-28 localized to nuclear membrane and cytoplasm with the C-terminal tag and nucleus and cytoplasm with the N-terminal tag. SIP2-28 was predicted as most likely to be nuclear with PsortII and equally likely

Copyright © 2004 John Wiley & Sons, Ltd. 

**Table 2.** The proteins were classified into three groups according to their subcellular localization under the heading 'Group'

| Gene Name | MGC ID | Group | Class | Length (AA) | Cell Localization (This Study) C-TERM | Cell Localization (This Study) N-TERM | Cell Localization (Previous Studies) Sw-Prot/Trembl | Cell Localization- Bioinformatic Prediction: PSORT II — OVER 20% | | Protcomp V.4 — Over 1 | LDB | PLDB | NN | ALL | Cell Localization Signal? Predict NIs | SW-PROT |
|---|---|---|---|---|---|---|---|---|---|---|---|---|---|---|---|---|
| ATF4 | 9337 | 1 | FACTOR | 352 | NUC | NUC | NUC | NUC | 74 | NUC | 18270 | 0 | 2.3 | 6.3 | NONE | NONE |
| CALM2 | 5226 | 1 | KINASE | 150 | NUC/CYTO | NUC/CYTO | NONE | CYTO | 52 | CYTO | 2315 | 0 | 2.1 | 2.6 | NONE | NONE |
| | | | | | | | | NUC | 22 | NUC | 0 | 0 | 2.3 | 2.3 | | |
| CDK7 | 5090 | 1 | KINASE | 347 | NUC/CYTO | NUC/CYTO | NUC | MITO | 39 | NUC | 18140 | 17265 | 1.7 | 7.7 | NONE | NONE |
| | | | | | | | | ER | 30 | CYTO | 4010 | 0 | 2.1 | 3 | | |
| CDK9 | 5166 | 1 | KINASE | 373 | NUC/CYTO | NUC/CYTO | NUC | CYTO | 65 | NUC | 19510 | 6030 | 2.1 | 7.1 | NONE | NONE |
| | | | | | | | | NUC | 26 | CYTO | 0 | 0 | 2.2 | 2.2 | | |
| IL17BR | 5245 | 1 | RECEPTOR | 503 | ER | ER | PM | ER | 44 | PM | 0 | 0 | 2.2 | 2.2 | NONE | N-TERM |
| | | | | | | | | GOLGI | 33 | EC | 0 | 0 | 2.1 | 2.1 | | |
| | | | | | | | | PM | 22 | | | | | | | |
| NFIB | 5146 | 1 | FACTOR | 421 | NUC | NUC | NUC | NUC | 78 | NUC | 22495 | 0 | 2.3 | 7.3 | NONE | NONE |
| PTPN11 | 17206 | 2 | RECEPTOR | 461 | CYTO | NUC/CYTO | CYTO | CYTO | 35 | CYTO | 24590 | 24360 | 1.6 | 9.8 | NONE | NONE |
| | | | | | | | | MITO | 26 | MITO | 0 | 0 | 1.8 | 1.8 | | |
| TNFR-SF10B | 5144 | 2 | FACTOR | 441 | TUB/VES | NUC | PM | ER | 26 | EC | 0 | 0 | 1.9 | 1.9 | NONE | N-TERM |
| | | | | | | | | PM | 26 | MITO | 0 | 0 | 1.2 | 1.2 | | |
| TGIF | 5066 | 2 | FACTOR | 273 | NUC/PUNCT CYTO | NUC/CYTO | NUC | NUC | 48 | NUC | 14130 | 0 | 2.3 | 5.4 | NONE | NONE |
| | | | | | | | | EC | 22 | | | | | | | |
| MARKL1 | 17739 | 2 | KINASE | 80 | MITO | NUC | NONE | MITO | 39 | PM | 1560 | 1220 | 0.9 | 1.4 | NONE | NONE |
| | | | | | | | | CYTO | 39 | MITO | 0 | 0 | 1.3 | 1.3 | | |
| | | | | | | | | | | EC | 0 | 0 | 1.3 | 1.3 | | |
| | | | | | | | | | | NUC | 1430 | 0 | 0.7 | 1 | | |
| SIP2-28 | 5148 | 2 | KINASE | 192 | NUC MEM/CYTO | NUC/CYTO | NONE | NUC | 70 | CYTO | 0 | 2430 | 2.3 | 2.6 | NONE | NONE |
| | | | | | | | | MITO | 30 | NUC | 0 | 0 | 2.2 | 2.2 | | |
| | | | | | | | | | | MITO | 0 | 0 | 1.2 | 1.2 | | |
| | | | | | | | | | | EC | 0 | 2040 | 0.9 | 1.1 | | |
| CXADR | 5086 | 3 | RECEPTOR | 366 | MV/SS | NONE | PM | EC | 35 | PM | 18950 | 0 | 2.3 | 6.6 | NONE | N-TERM (POTENTIAL) |
| | | | | | | | | PM | 30 | EC | 0 | 0 | 2.3 | 2.3 | | |
| | | | | | | | | ER | 22 | PEROX | 0 | 0 | 1.1 | 1.1 | | |
| NFIL3 | 5038 | 3 | FACTOR | 463 | NUC | NONE | NONE | NUC | 87 | NUC | 0 | 0 | 2.3 | 2.3 | N-TERM | NONE |
| PPARG | 5041 | 3 | RECEPTOR | 478 | NUC | NONE | NUC | CYTO | 65 | NUC | 24700 | 7180 | 2.4 | 8.6 | N-TERM | NONE |
| | | | | | | | | NUC | 22 | CYTO | 0 | 0 | 1.7 | 1.7 | | |
| STK15 | 5133 | 3 | KINASE | 404 | NUC | NONE | CENT/SP (NUC) | NUC | 78 | NUC | 4350 | 0 | 2.2 | 3.2 | NONE | NONE |
| | | | | | | | | | | CYTO | 4220 | 4375 | 0.8 | 2.3 | | |
| | | | | | | | | | | ER | 0 | 0 | 1.2 | 1.2 | | |
| CDKN1B | 5304 | 3 | KINASE | 199 | NUC | NONE | NUC | NUC | 78 | NUC | 10565 | 9830 | 2.3 | 5.7 | NONE | C-TERM (POTENTIAL) |

Group 1, same subcellular localizations with N- and C-terminal tagged GFP fusion proteins; group 2, differing subcellular localizations with N- and C-terminal tagged GFP proteins; group 3, subcellular localizations observed with C-terminal-tagged, but not with N-terminal-tagged, GFP proteins. Our subcellular localizations, previous Swiss-Prot/TrEMBL localizations and Psort II and ProtComp 4 localization predictions were recorded. Signal peptide locations were recorded using PredictNLS and Swiss-Prot/TrEMBL. Light grey boxes indicate equivalent subcellular compartments between the subcellular localizations from this study and previous and predicted subcellular localizations. Dark grey boxes indicate signal sequences. AA, amino acids; CENT, centrosome; CYTO, cytoplasm; EC, extra cellular; ER, endoplasmic reticulum; LYSO, lysosome; MITO, mitochondria; MV, microvilli; NUC, nucleus; NUC MEM, nuclear membrane; PEROX, peroxisome; PM, plasma membrane; PUNCT CYTO, punctate cytoplasm; SP, spindle pole; SS, surface structures; TUB, tubule; VES, vesicles; NLS, nuclear localization signal; C-TERM, C-terminal-tagged GFP; N-TERM, N-terminal-tagged GFP; SW-PROT, Swiss-Prot/TrEMBL; LDB, localization from database; PLDB, predicted location from database; NN, neural networks; ALL, combined results from LDB, PLDB and NN.

Copyright © 2004 John Wiley & Sons, Ltd.

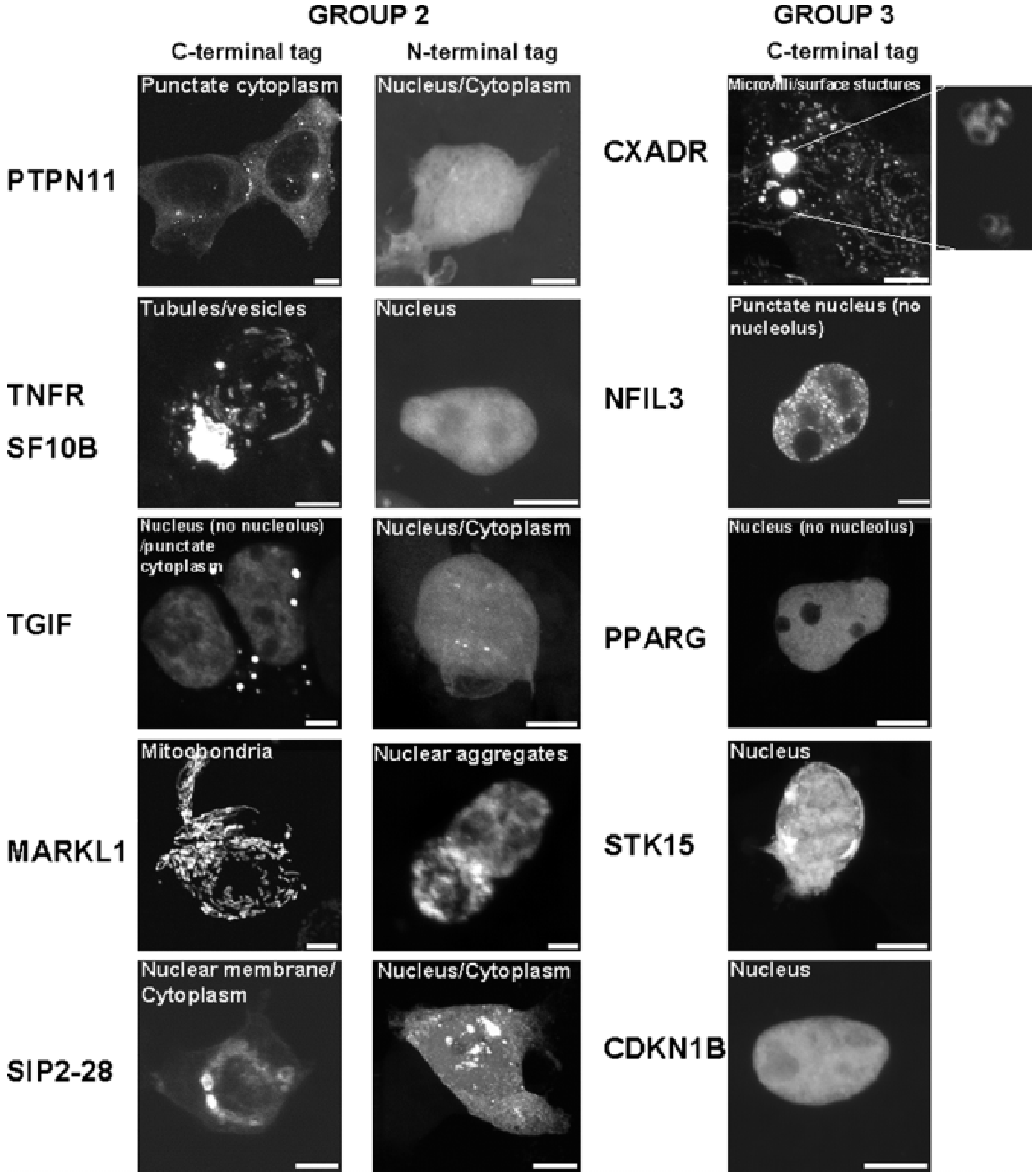

**Figure 3.** Group 2, C and N-terminal tagged proteins with differing subcellular localizations. Group 3, C-terminal tagged protein subcellular localizations (N-terminal tagged proteins did not localize). A confocal microscope with a ×60 oil lens was used to obtain 16 slices through the cell for each image. All images are of the whole cell except TGIF, NFIL3 and PPARG, which are slices to demonstrate the empty nucleoli, and SIP2-28, which is a slice to demonstrate the empty nucleus. Bar, 10 μm to be nucleus or cytoplasm with ProtComp version 4; it is therefore difficult to determine which is the correct localization for SIP2-28, as the results are so similar.

Five genes (PTPN11, TNFRSF10B, TGIF, MARKL1 and SIP2-28: group 2; Figure 2 and Table 2) differed in localization when tagged with GFP at the N- or C-terminal end. C-terminal-tagged PTPN11 localized to the cytoplasm and N-terminal to the nucleus/cytoplasm in this study. The Swiss-Prot/TrEMBL localization was cytoplasmic and both bioinformatics studies predicted the cytoplasm.

Copyright © 2004 John Wiley & Sons, Ltd.

C-terminal-tagged TNFRSF10B localized to tubules/vesicles and N-terminal to the nucleus, the Swiss-Prot/TrEMBL localization was the plasma membrane, Psort II predicted the endoplasmic reticulum and plasma membrane as equally likely localizations and the ProtComp version 4 prediction was extracellular. C-terminal-tagged TGIF localized to the nucleus, with small punctate bodies within the cytoplasm and N-terminal to the nucleus/cytoplasm. The Swiss-Prot/TrEMBL localization is nuclear and both programmes predicted the nucleus. C-terminal tagged MARKL1 localized to mitochondria and N-terminal to the nucleus, the mitochondria was predicted as a highly likely localization by both programmes. In each case the C-terminal localization was more likely to be correct. SIP2-28 was observed as in the previous paragraph.

N-terminal tagged group 3 proteins showed no expression at all and N-terminal tagged group 2 proteins show a different localization when tagged at the C-terminal (Figure 3, Table 2). The C-terminal localizations were concluded to be more likely to be correct in each case, after comparison (above) with previous subcellular localization studies and results from bioinformatic predictions. The GFP tagging of the GOI at the N-terminal end would therefore appear to be having a detrimental effect on protein localization. A possible cause could be the location of the signal sequence, which is more often at the N-terminal end of the protein.

In order to examine the proteins for the presence of a protein-sorting sequence, Swiss-Prot (Boeckmann, 2003) and the PredictNLS programme (Cokol, 2000) were used. Swiss-Prot records any known targeting sequences from the literature; if none are found, the sequence is analysed using the programme Signal P (Neilsen, 1997). Signal P is based on neural networks trained on separate sets of eukaryote and prokaryote sequences, if a signal peptide is predicted it is recorded in Swiss-Prot as 'potential'. PredictNLS checks the protein sequence for a match to 214 potential nuclear localization signals (NLS). The programme only predicts signal peptides for localization to the nucleus, but was chosen since we found eight of the 16 proteins in this study to localize to the nucleus with the N- or C-terminal tag. Six predicted targeting sequences were found in the 16 proteins (Table 2). An N-terminal signal sequence was discovered in three group 3 proteins (CXADR, NFIL3 and PPARG), one group 2 protein (TNFRSF10B) and one group 1 protein (IL17BR). A single C-terminal signal sequence was predicted in the group 3 protein CDKN1B.

### Discussion

Reverse transfection arrays have the potential to provide a high throughput means of screening for gene function. Our aim was to explore their utility as a method for determining the subcellular localization of proteins. In this pilot study, 16 genes from a variety of functional classes that had different subcellular localizations were chosen, in order to ensure that the findings would be applicable to a variety of different gene classes. Their ORFs were amplified from full-length MGC cDNA clones and inserted into N- and C-terminal GFP Gateway expression vectors. The expression constructs were then packaged in transfection reagent and printed onto a glass slide to form a microarray. HEK293T cells were grown on top of the slide until confluent and patches of transfected cells overexpressing the genes were formed. GFP was chosen to tag the ORFs, as it can be visualized easily via confocal microscopy, with no further need for manipulation of the arrays. Due to the relatively large size of GFP, N- and C-terminal fusions were constructed to explore the optimal position of the tag with respect to normal localizations of the protein.

In some cases transfection efficiencies were comparable with those reported by Sabatini and Ziauddin, with 30–80 of cells transfected per spot. However, they varied greatly in this study, from as little as 5% to more than 80% of spots transfected per protein, with 2–30 cells transfected per spot. The variation in transfection efficiency could be due to the number of gene copies within the cell, the rate of gene transcription and the stability of the mRNA transcript (Colosimo, 2000). In categorizing and determining whether the observed subcellular localizations were correct, Swiss-Prot/TrEMBL, Psort II and Protcomp 4 were very useful. Swiss-Prot/TrEMBL records any previous subcellular localizations of proteins reported in the literature, Psort II and Protcomp 4 predict localizations. Subcellular localizations for 12 of the 16 proteins studied here were recorded in Swiss-Prot/TrEMBL and novel subcellular localizations were observed for the remaining four (NFIL3, CALM2, MARKL1 and SIP2-28).

Copyright © 2004 John Wiley & Sons, Ltd. 

NFIL3 is a transcription factor, which explains its occurrence in the nucleus in this study. CALM2, together with the proteins CALM1 and CALM3 form the protein calmodulin (Tootenhoofd, 1998); we found CALM2 equally distributed throughout the nucleus and cytoplasm. MARKL1 was found in the mitochondria, there is little other information available on this gene. SIP2-28 is a calcium binding protein, which binds to the cytoplasmic domains of integrin $\alpha$IIb; in this study it localized to the nucleus/nuclear membrane and the cytoplasm. These subcellular localizations were all supported by the bioinformatics prediction programmes (Table 2). This study, however, is too small to draw any conclusions about the general accuracy of protein localization prediction programmes.

All 16 of the C-terminal tagged proteins localized correctly, but this was the case for less than half of the N-terminal tagged proteins. N-terminal signal peptides were found in the group 2 and 3 proteins TNFRSF10B, CXADR, NFIL3 and PPARG (these groups contained N-terminal-tagged proteins that either did not localize correctly or did not show any protein expression). This supports evidence that N-terminal GFP tagging of a protein can cause the signal sequence at the N-terminus to be masked. The lack of expression or mislocalization of the N-terminal tagged proteins could possibly be explained further. Proteins emerge from the ribosome into the cytoplasm N-terminus first and chaperones prevent the amino acid chain from folding until a whole domain, 50–300 amino acids long, has emerged (Hartl, 2002). GFP is 238 amino acids long and will fold first if tagged at the N-terminal end, possibly disrupting further folding and correct localization of the protein, regardless of whether its signal sequence is at the N- or C-terminal end. In some cases the protein may disrupt the folding of the GFP itself, giving rise to the observation of group 3 proteins, i.e. no visible protein expression. C-terminal tagging of the protein with GFP would not have the same effect, as the GFP will be folded last and will not influence the native or GFP protein conformation. Overall, this study suggests that C-terminal tagging of a protein with GFP is generally superior to N-terminal tagging, as the protein is more likely to localize correctly and therefore it is to be expected that it will maintain the functional characteristics of the native protein.

A number of groups have recently reported results from large-scale subcellular localization studies using 96-well plates (Wiemann, 2003; Huh, 2003). Weimann *et al.* performed subcellular localization studies on over 500 C- and N-terminal fluorescently tagged human genes from the German cDNA consortium collection (Weimann, 2001). Huh *et al.* localized 6029 C-terminal GFP tagged ORFs from *Saccharomyces cerevisiae.* Weimann *et al.* concluded that most signal peptides located at the N-terminus of proteins, such as those that direct proteins to the mitochondria and plasma membrane, were masked by the N-terminal GFP fusion, as our study also demonstrates (see group 2 proteins; Figure 3). Huh *et al.* tagged their ORFs at the C-terminus only; 80% of their subcellular localizations were in agreement with previous findings, but they concluded that proteins localized to the cell wall, peroxisome and ER, which often contain C-terminal targeting signals, were mislocalized due to the C-terminal GFP. Therefore, both these large-scale studies and the pilot study described here are in agreement — the majority of C-terminal tagged ORFs localize correctly, as most signal peptides are at the N-terminus, but it is preferable to tag at both ends, as some signal peptides are found at the C-terminus.

In theory, reverse transfection has an advantage over plate-based assays, in that more plasmids can be transfected simultaneously. However, this study has exposed some of the inherent challenges in setting up this technology as a high-throughput system. Whilst large collections of sequence-verified full-length cDNA clones are available via the MGC (Strausberg, 2002), the Full Length Expression (FLEX) repository initiative (Brizuela, 2002) and the German cDNA consortium (Weimann, 2001), these genes are not tagged. Sub-cloning of the ORFs into the Gateway cloning system is relatively expensive, time-consuming and could potentially introduce errors into the ORFs; therefore, ideally, the clones need to be resequenced. Another issue is imaging these arrays. A high-throughput imaging system would undoubtedly be needed to systematically record the subcellular localizations of proteins and/or read-outs of downstream assays if any number of genes/arrays were to be processed at any time. The necessity for spot recognition software and the storage and analysis of the images present a considerable challenge. These issues are currently being explored and a microscope-based screening

Copyright © 2004 John Wiley & Sons, Ltd. 

platform is being developed with automated sample preparation, image acquisition and data analysis (Wiemann, 2003; Liebel, 2003). If these challenges can be overcome, reverse transfection technology could prove to be an enormously powerful tool for the characterization of gene function.

### Acknowledgements

Thanks to Chris Sanderson for critical reading of the manuscript and assistance with subcellular localizations, and Jayn Wright for help with microarray printing. This work was funded by the MRC.

### References

Bailey SN, Wu RZ, Sabatini DM. 2002. Applications of transfected cell microarrays in high-throughput drug discovery. *Drug Discov Today* **7**(suppl): S113–118.

Boeckmann B, Bairoch A, Apweiler R, *et al*. 2003. The Swiss-Prot protein knowledgebase and its supplement Trembl in 2003. *Nucleic Acids Res* **31**: 365–370.

Brizuela L, Richardson A, Marsischky G, Labaer J. 2002. The Flexgene Repository: exploiting the fruits of the genome projects by creating a needed resource to face the challenges of the post-genomic era. *Arch Med Res* **33**: 318–324.

Chester N, Marshak DR. 1993. Dimethyl sulfoxide-mediated primer Tm reduction: a method for analyzing the role of renaturation temperature in the polymerase chain reaction. *Ann Biochem* **209**: 284–290.

Cokol M, Nair R, Rost B. 2000. Finding nuclear localization signals. *EMBO Rep* **1**: 411–415.

Colosimo A, Goncz K, Holmes A, *et al*. 2000. Transfer and expression of foreign genes in mammalian cells. *Biotechniques* **29**: 314–331.

Hartl F, Hayer-Hartl M. 2002. Molecular chaperones in the cytosol: from nascent chain to folded protein. *Science* **295**: 1852–1858.

Huh W, Falvo J, Gerke LC, *et al*. 2003. Global analysis of protein localization in budding yeast. *Nature* **425**: 686–691.

Liebel U, Starkuviene V, Erfle H, *et al*. 2003. A microscope-based screening platform for large-scale functional protein analysis in intact cells. *FEBS Lett* **554**: 394–398.

Lippincot-Schwartz J, Patterson GH. 2003. Development and use of fluorescent protein markers in living cells. *Science* **300**: 87–91.

Nakai K. 2001. Review: prediction of *in vivo* fates of proteins in the era of genomics and proteomics. *J Struct Biol* **134**: 103–116.

Nakai K, Kanehisa M. 1991. Expert system for predicting protein localization sites in Gram-negative bacteria. *Proteins* **11**: 95–110.

Nakai K, Kanehisa M. 1992. A knowledge base for predicting protein localization sites in eukaryotic cells. *Genomics* **14**: 897–911.

Nakai K, Norton P. 1999. Psort: a programme for detecting sorting signals in proteins and predicting their subcellular localization. *Trends Biochem Sci* **24**: 34–35.

Neilsen H, Engelbrecht J, Brunak S, von Heijne G. 1997. Identification of prokaryotic and eukaryotic signal peptides and prediction of their cleavage sites. *Protein Eng* **10**: 1–6.

Strausberg RL, Feingold EA, Grouse LH, *et al*. 2002. Generation and initial analysis of more than 15 000 full-length human and mouse cDNA sequences. *Proc Natl Acad Sci USA* **99**: 16 899–16 903.

Tootenhoofd SL, Foletti D, Wicki R, *et al*. 1998. Characterization of the human CALM2 calmodulin gene and comparison of the transcriptional activity of CALM1, CALM2 and CALM3. *Cell Calcium* **23**: 323–338.

Weimann S, Weil B, Wellenreuther R, *et al*. 2001. Toward a catalog of human genes and proteins: sequencing and analysis of 500 novel complete protein coding human cDNAs. *Genome Res* **11**: 422–435.

Wiemann S, Bechtel S, Bannasch D, *et al*. 2003. The German cDNA network: cDNAs, functional genomics and proteomics. *J Struct Funct Genom* **4**: 87–96.

Wu RZ, Bailey SN, Sabatini DM. 2002. Cell-biological applications of transfected-cell microarrays. *Trends Cell Biol* **12**: 485–488.

Ziauddin J, Sabatini DM. 2001. Microarrays of cells expressing defined cDNAs. *Nature* **411**: 107–110.

Copyright © 2004 John Wiley & Sons, Ltd. 
